# Evaluation of age-related thymic changes using computed tomography images: A retrospective observational study

**DOI:** 10.1097/MD.0000000000029950

**Published:** 2022-08-12

**Authors:** Kosuke Suzuki, Akihiko Kitami, Momoka Okada, Shinnosuke Takamiya, Shinichi Ohashi, Yoko Tanaka, Syugo Uematsu, Mitsutaka Kadokura, Takashi Suzuki, Norihiro Hashizume, Hidefumi Fujisawa

**Affiliations:** a Respiratory Disease Center, Showa University Northern Yokohama Hospital, Yokohama, Japan; b Department of Thoracic Surgery, Showa University Fujigaoka Hospital, Yokohama, Japan; c Department of Radiology, Showa University Northern Yokohama Hospital, Yokohama, Japan.

**Keywords:** adipose tissue, age, computed tomography, thymus

## Abstract

We aimed to investigate if Computed tomography (CT) attenuation values can help improve the identification of age-related changes in the thymus.

We assessed CT images of 405 patients aged 0 to 80 years. We measured the area of the anterior mediastinum at the level of the carina and its average CT attenuation value. We evaluated the thymic area, the ratio of the thymus area to the total thoracic area, and the CT attenuation value. Additionally, we evaluated changes in the thymus area in the 0 to 13-year age group.

The area of the thymus decreased from birth to the middle 20s. After the middle 20s, the area tended to increase and plateau till after 50 years of age. The ratio of the thymic area to the thoracic area decreased from age 0 to 20 years, but remained stable after 20 years of age. The CT attenuation values were stable from birth to puberty, decreased after puberty, and were stable again in the late 50s and beyond. The thymus of children showed mass formation, but the shape changed with age. No significant differences in the CT attenuation value were found across underlying conditions for the 0 to 13-year age group.

The decrease in the CT attenuation values, observed with advancing age, reflects adipose degeneration of the thymus, indicating that by the late 50s, thymic tissue is replaced completely by adipose tissue. Our data suggest that adipose degeneration of the thymus begins after puberty and advances with age.

## 1. Introduction

The thymus gland is important in T-cell production and maturation; however, the production of T-cells in the thymus decreases after puberty. The weight of the thymus becomes maximal at puberty, after which it decreases in size and undergoes adipose degeneration until it has been replaced completely by adipose tissue.^[1]^

Using CT images, it is not always trouble-free to distinguish between the enlarged thymus and mediastinal tumors in infants, and between the remnant thymus and thymic tumors in adults.

We assessed changes in the thymus gland related to age using CT images, and demonstrated various shapes of the thymus using CT data from children.

## 2. Methods

We retrospectively reviewed CT images of the thorax of 405 patients who had no lesion or injury in mediastinum (n = 5 patients for each age) between 0 and 80 years of age, from the Showa University Northern Yokohama Hospital records. Exclusion criteria included advanced deformation of the thorax, endocrine disease, autoimmune disease, and post median sternotomy.

We measured the area of the anterior mediastinum at the level of the carina, as well as its average CT attenuation value. Concerning the area measurements, we evaluated not only the thymic area itself but also the ratio of the thymic area to the total thoracic area at the same CT slice level. Regarding the CT attenuation value, we also evaluated the differences between males and females. Additionally, we classified 4 types of thymus shapes: large, flat, sharp, and small types, and evaluated the proportion of each type in 70 patients younger than 13 years. Furthermore, we compared the average CT attenuation values, grouped by the underlying condition at the time of CT examination, for the 0 to 13-year age group.

CT was performed using an Aquilion ONE, Aquilion 64 (Toshiba Medical Systems) and SOMATOM Definition Flash (Siemens). The slice thickness was 2 mm at 120 kV, taken at the level of the carina. We created regions of interest (ROIs) by tracing the anterior mediastinal tissue using the image reference viewer (HOPE/Dr ABLE-EX, Fujitsu, Tokyo, Japan) of the electronic medical record system (HOPE/EGMAIN-FX, Fujitsu, Tokyo, Japan). The ROIs were evaluated for the area (in mm^2^) and average CT attenuation value (in Hounsfield Units, HU).

**Figure 1.**
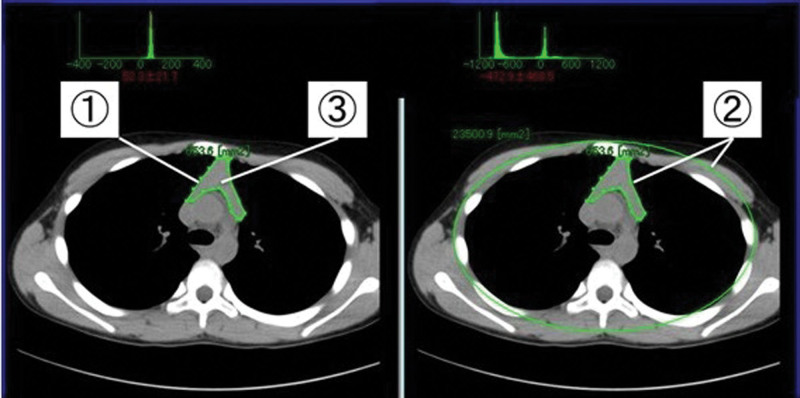
The method of creating ROIs. ROIs were created by tracing the anterior mediastinal tissue using the image reference viewer. The ROIs were evaluated for the area (①) and average CT attenuation value (③). The ratio of the ROI area with the total thoracic area was also calculated using the area of an ellipse drawn around the bilateral ribs, sternum, and vertebrae at the level of the carina to represent the total thoracic area (②).

The ratio of the area of the ROI to the total thoracic area was calculated using the area of an ellipse drawn around the bilateral ribs, sternum, and vertebrae at the level of the carina to represent the total thoracic area (Fig. 1).

Statistical evaluation was performed using JMP Pro 15.0.0. (SAS). In scatter plot diagrams for each factor, we drew the regression line, and calculated the R2. We evaluated the CT attenuation values grouped by the underlying conditions for the 0 to 13-year age group, using nonparametric Kruskal–Wallis test. The level of significance was set at *P* ≤ .05.

The Showa University Northern Yokohama Hospital Ethics Committee approved this study.

## 3. Results

The 405 patients included 237 males and 168 females, on whom CT was performed to evaluate (1) an abnormal appearance of chest X-P or screen (122 cases, 30%), (2) viral infection (70 cases, 17%), and (3) pneumothorax (55 cases, 13%) (Table 1).

**Table 1:** The purpose of taking CT images.

	*N*
Abnormal appearance or screen	122
Viral infection	70
Pneumothorax	55
Tumor	35
Asthma or dyspnea	40
Interstitial pneumonia or emphysema	24
Trauma	6
Thorax abnormality	5
Convulsion or disturbance of consciousness	4
Aspiration or accidental ingestion	4
Other	40

**Figure 2.**
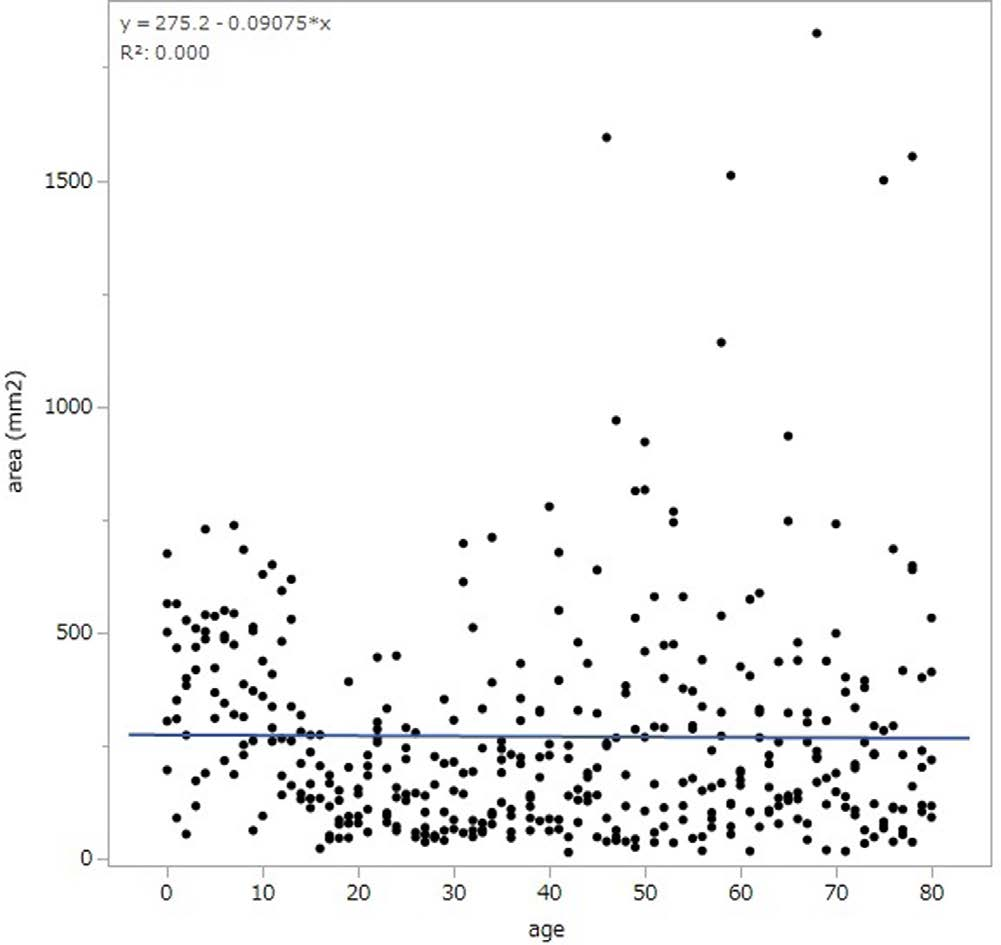
Area of anterior mediastinal tissue. The area decreased from birth to the mid-20s. After the mid-20s, the area tended to increase and plateau until after the age of 50 years.

The area of the anterior mediastinal tissue varied slightly in each individual. The area decreased from birth to the middle 20s. After the middle 20s, the area tended to increase and plateau till after 50 years of age (Fig. 2).

**Figure 3.**
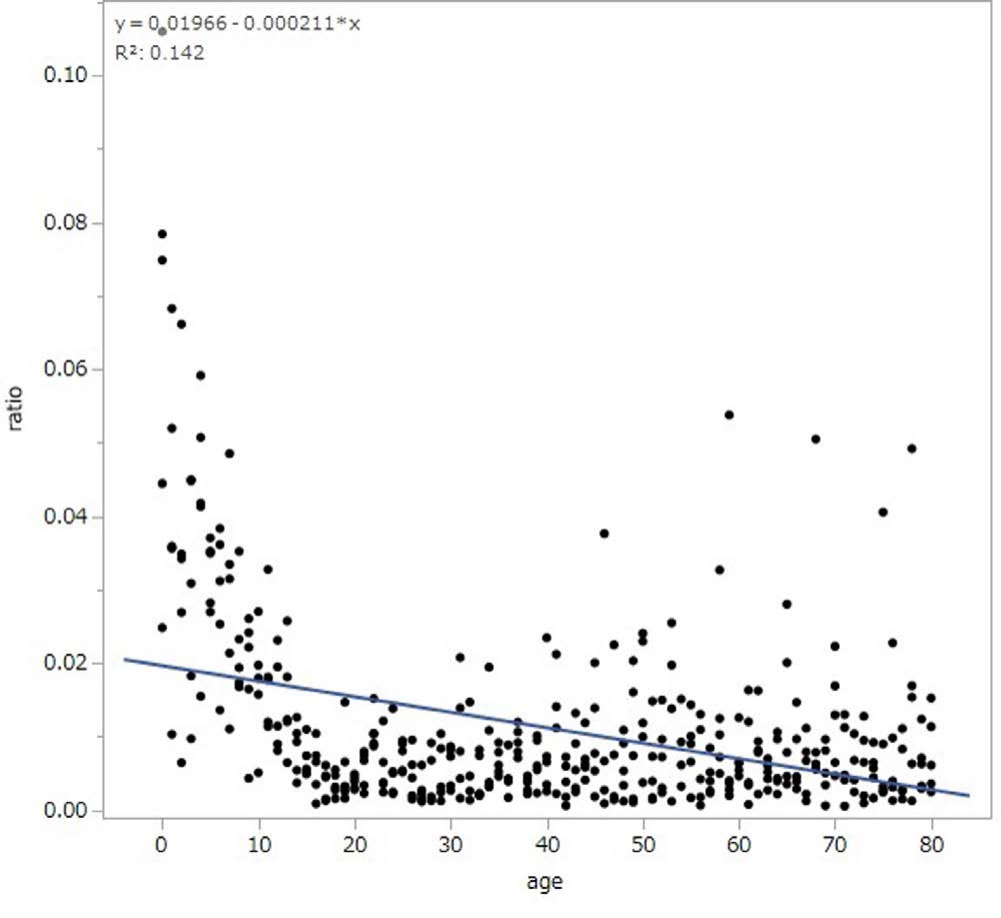
The ratio of the anterior mediastinal tissue/thorax. The anterior mediastinum-to-thorax ratio areas showed large interindividual differences in infants, which grew smaller with age. The mean ratio area decreased with age from 0 to 20 years but was stable after the age of 20 years.

The area ratio of the anterior mediastinum-to-thorax showed large interindividual differences in infants, which grew smaller with age. The area mean ratio decreased with age from 0 to 20 years, but was stable after 20 years (Fig. 3).

**Figure 4.**
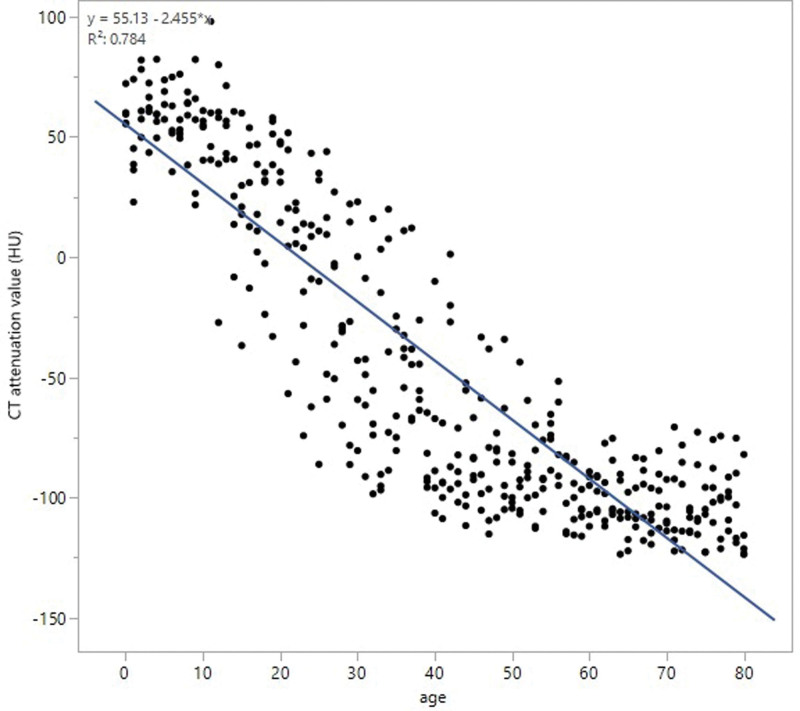
CT attenuation value. The average CT attenuation value was 60.5 ± 6.8 HU at the age of 0 year and − 102.4 ± 13.2 HU in the late 50s or beyond. The average CT attenuation value was not significantly different up to the age of 13 years. From the age of approximately 13 years to the late 50s, the average CT attenuation value decreased. The average CT attenuation value was not significantly different after the late 50s.

**Figure 5.**
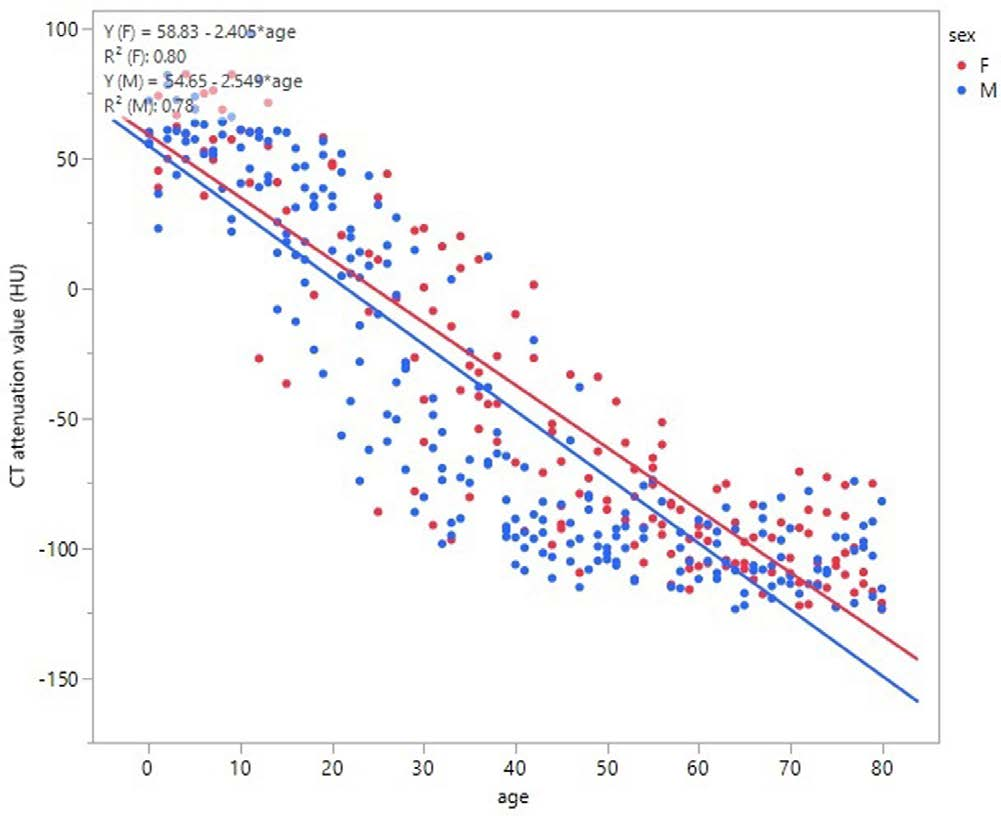
CT attenuation value (male and female). The average CT attenuation value per decade of age did not show any significant statistical difference between males and females (*P* = .5995); however, it declined faster in males than in females.

The average CT attenuation value was 60.5 ± 6.8 HU at 0 years of age, which was not significantly different up to the age of 13 years (56.4 ± 17.6 HU). From approximately 13 to 20 years of age, the average CT attenuation value decreased, and in the early 20s, the average CT attenuation value became under 0 HU. In the late 20s to late 30s years of age, it decreased further to − 30 to − 50 HU. Patients in their late 50s or later showed an average CT attenuation value of − 102.4 ± 13.2 HU. The overall average CT attenuation value for patients aged 14–56 years was − 42.3 ± 50.3 HU (Fig. 4). The average CT attenuation value per decade of age showed no significant statistical difference between males and females (*P* = .5995), but it declined faster in males than in females (Fig. 5).

**Figure 6.**
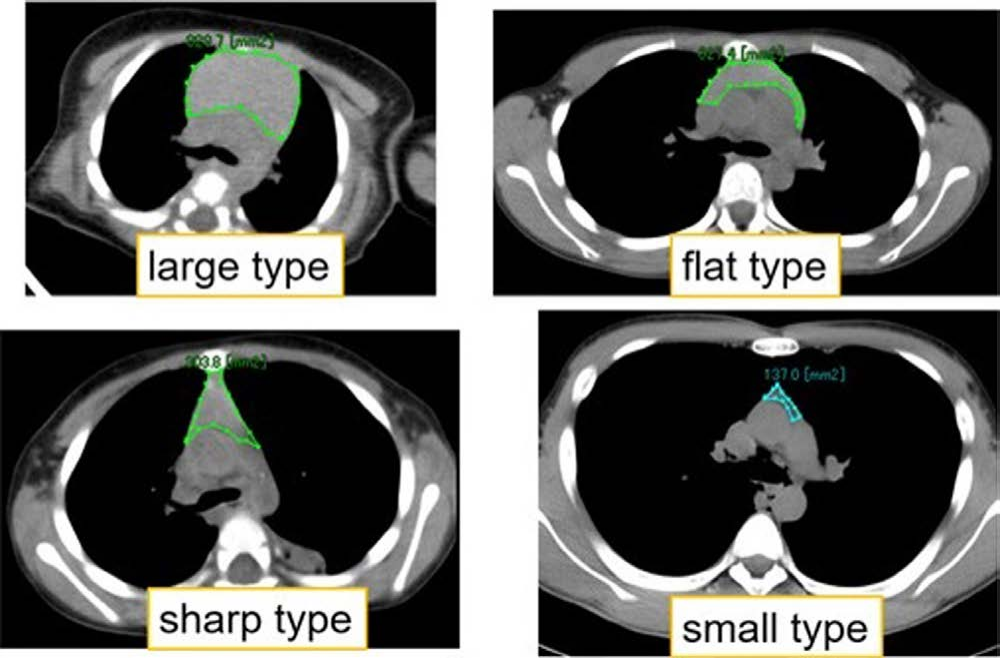
The types of thymus in children classified into 4 types of thymus shapes.

We recognized large mass formation of the thymus in some patients at a younger age, and features of various shape types in each generation under 13 years. We classified up to 4 types of thymus shapes: “large type,” which has a round shape resembling a tumor; “flat type,” which is shortened in the anteroposterior direction “sharp type,” which is shortened laterally; and the “small type,” which is shortened in both anteroposterior and lateral direction (Fig. 6). At 0 years of age, 60% of patients had a large type of thymus, the size of which decreased with age. The percentages of flat and sharp types increased with age, whereas around puberty, the small type was observed (Table 2).

**Table 2:** The shape of the thymus in the 0 to 13-year age group.

Age (yr)	Large (*N*)	Flat (*N*)	Sharp (*N*)	Small (*N*)
0	3	1	1	0
1	2	2	1	0
2	2	1	2	0
3	2	2	1	0
4	1	3	1	0
5	1	4	0	0
6	1	3	1	0
7	1	2	2	0
8	0	4	1	0
9	0	2	2	1
10	0	0	4	1
11	0	2	3	0
12	0	0	4	1
13	0	2	3	0

To evaluate differences in the CT attenuation value by the underlying conditions, we compared the CT attenuation values, grouped by condition, for the 0 to 13-year age group. No significant differences were found across any conditions by Kruskal–Wallis test (*P* = .9539) (Table 3).

**Table 3:** CT attenuation value in the 0 to 13-year age group classified by an underlying condition.

Underlying condition	*N*	Median CT attenuation value (HU)	Interquartile range
Infection	28	60.25	24.02
Asthma/wheezing	11	57.2	22.8
Convulsions/disturbance of consciousness	4	60.85	15.1
Deformation of thorax	4	64.3	18.05
Aspiration/accidental ingestion	3	57.4	36.8
Trauma	3	56.6	34.2
Others	17	57.2	13.7

## 4. Discussion

The thymus is the primary lymphoid organ in the anterior mediastinum; it is visualized as a butterfly-shaped region of nonadipose or adipose tissue. Functionally, it participates in the production and maturation of T-cells. The thymic tissue is dense from infancy through puberty, after which it decreases in size and undergoes adipose degeneration.^[1]^ These changes continue with advancing age, such that the thymus tissue is replaced by adipose tissue in adults. There are several reports that review normal thymic appearance or thymic changes on CT images with age,^[2–5]^ but there are hardly any evaluations of CT appearance from birth to older age. In this study, we attempted to assess age-related changes in the thymus based on CT images and their attenuation values.

We checked CT images at the level of the carina, as the boundary between the anterior mediastinal tissue and the surrounding structure is clear. We noted that the area of the anterior mediastinal tissue was similar for ages 0 to 13 and 14–20 years. The area ratio decreased from birth to the early 20s. This effect is related to the stable size of the thymus and the growth of the thoracic area during these years. This finding correlates with reports describing the thymus as decreasing in size from birth onward.^[6]^

The mean CT attenuation value was approximately 60 HU from birth to puberty and continued to decrease to about −100 HU (equivalent to pure adipose tissue) by the age of the late 50s. Drabkin et al^[7]^ reported increased fatty replacement of the thymus occurred with increasing age in their evaluation of adult multidetector CT findings. In this study, we confirm that the decrease in CT attenuation values from puberty to the 20s reflects the decreased density of the anterior mediastinal tissue, resulting from ongoing adipose degeneration.

After 20 years of age, the area of the anterior mediastinal tissue and area ratio showed interindividual differences, but the average ratio was generally stable. Therefore, after the age of 20 years, thymic tissue undergoes significant adipose degeneration, and by the late 50s, it degenerates completely to adipose tissue. Shimanovsky et al^[5]^ evaluated the CT appearance of the thymus in patients with trauma, and the volume and density of the thymus declined from puberty to 55 years of age, which is the same as in our study.

Thymectomy is performed in myasthenia gravis (MG). According to international consensus guidance,^[8]^ all patients with MG with thymoma should undergo surgery to remove the tumor. In nonthymomatous MG, however, thymectomy is performed as an option to avoid or minimize the dose or duration of immunotherapy, or if patients fail to respond to an initial trial of immunotherapy or have intolerable side-effects from that therapy. Gronthes et al^[9]^ reported that the efficacy of thymectomy to nonthymomatous MG is better in younger patients than in patients of advanced age. On the other hand, in a MGTX trial,^[10]^ which is a randomized study of thymectomy in MG, the efficacy of thymectomy is shown both in participants with disease onset at age younger than 40 years and in those with disease onset at 40 years or older. It should be noted that thymectomy to treat nonthymomatous MG is most effective in younger patients. However, a positive effect of thymectomy in patients of advanced age with nonthymomatous MG could occur as a result of nondegenerated thymic tissue being extracted in the resected (but mostly degenerated) specimen. In this study, the low average CT attenuation value, which was the same as that of pure adipose tissue after the late 50s, seems to represent the high proportion of the degenerated part of the thymus and the low proportion of thymic tissue. We are interested in the correlation between the CT attenuation value and the efficacy of thymectomy to nonthymomatous MG. We conclude that further investigation is needed.

Rezzani et al^[11]^ mentioned that the acceleration of thymic decline after puberty is more pronounced in males than in females, suggesting that increasing levels of sex hormones contribute to the involution process of the thymus. Thapa et al^[12]^ describe that thymic involution occurs at a faster rate in males than in females, suggesting a role of androgens in thymic atrophy. Similarly, in our study, the CT attenuation value in males decreased faster than that in females, but there was no statistically significant difference.

From birth to puberty, the thymus tissue is dense. Sklair-Levy et al^[2]^ evaluated the CT attenuation value in children. In their study, in infants younger than 1 year of age, the mean thymic attenuation value was 80.8 ± 5.7 HU. This value is close to our outcome (60.5 ± 6.8 HU at 0 years of age). In addition to the CT attenuation value, we checked the change of shape of the thymus. At 0 years of age, the thymus was classified as a large type and resembled a tumor. The shape changes to a flat and sharp type with age. Around puberty, the small type of thymus shape appeared. Concerning the change of the anterior mediastinal area and the CT attenuation value, the shape types of the thymus appear to change as the size of thymus changes slightly. In contrast, the thoracic area becomes larger in the anteroposterior and lateral diameter with growth.

The thymus can be affected by several conditions. Thymic involution is caused by stress as well as age.^[1]^ Ansari et al^[13]^ demonstrated that acute thymic involution is caused by several infectious agents (bacteria, viruses, parasites, fungi) and other factors, including stress, pregnancy, malnutrition, and chemotherapy. Fukunaga et al^[14]^ showed that in most abused and/or neglected children, the weight of the thymus decreased conspicuously. Hanson et al^[15]^ reported that the shape of the thymus is influenced by Cushing syndrome, and 33% of patients with active Cushing syndrome showed significant thymic soft tissue remnant structures in the anterior mediastinum on CT images. In this study, we excluded those underlying conditions that could have chronic impacts on the thymus (e.g., endocrine disorders or physical interventions that can cause the deformation of the thorax). We also compared CT attenuation values in the 0 to 13-year age group, because they had the densest thymic tissues. Notably, Miriam et al^[2]^ reported that the density of the thymus tissue declines in the presence of malignant diseases in children aged <1 year, but not in the presence of acute or benign diseases. Our data support this observation, given that there were no significant differences in CT attenuation values by condition, although all the included conditions were acute diseases.

A limitation of this study was the retrospective nature of the study; the CT records may not have genuinely represented normative thymus results. It is possible that undiagnosed or undocumented conditions were present. In addition, Araki et al^[3]^ showed that cigarette smoking and a high body mass index (BMI) were associated with fatty degeneration of the thymic gland. Also, Harrington et al^[16]^ reported that in patients aged between 20 and 30 years, the degree of fatty infiltration of the thymus is related to the BMI. Overweight patients are more likely to have higher levels of thymic fatty infiltration than those in normal or underweight patients. However, in our evaluation, patients’ body weight, body length, BMI, smoking history, and so on were not taken into account. Moreover, this study was performed using the image reference viewer of our electronic medical record system, rather than Digital Imaging and Communications in Medicine (DICOM) data, because of time constraints. Therefore, it was difficult to reconstitute the image, making volume calculations less reliable. Furthermore, only 1 image level (slice) was used; hence, individual differences in the positioning of the thymus in the thorax might have affected our calculations. Finally, the influence of respiration during slice collection was not evaluated, which could also affect the area ratio calculations. On evaluating the CT attenuation value in the 0 to 13-year age group classified by the underlying conditions, there was imbalance in each condition. Therefore, our study may be affected by the possibility of differences in these conditions.

In this study, we examined age-related thymic changes using CT images, specifically analyzing CT attenuation values and ROIs. Our data suggest that there are minimal changes from birth to puberty, and the thymus has high density. However, after puberty, the thymus undergoes long-term adipose degeneration, and decreases in size and density with advancing age, until it is finally replaced completely by adipose tissue.

## Acknowledgments

The authors would like to thank Enago (www.enago.jp) for the English language review.

## Author contributions

KS and AK designed the study. KS collected and analyzed the data and drafted the manuscript. AK, MK, TS, NH, and HF advised on the study design and data analysis. All authors read and approved the final manuscript.
